# An Extract of Chinpi, the Dried Peel of the Citrus Fruit Unshiu, Enhances Axonal Remyelination via Promoting the Proliferation of Oligodendrocyte Progenitor Cells

**DOI:** 10.1155/2016/8692698

**Published:** 2016-02-28

**Authors:** Hideaki Tokunaga, Chika Seiwa, Nozomu Yoshioka, Kazushige Mizoguchi, Masahiro Yamamoto, Hiroaki Asou, Sadakazu Aiso

**Affiliations:** ^1^Center for Kampo Medicine, School of Medicine, Keio University, 35 Shinanomachi, Shinjuku-ku, Tokyo 160-8582, Japan; ^2^Department of Brain Development and Neural Regeneration, Tokyo Metropolitan Institute of Medical Science, 2-1-6 Kamikitazawa, Setagaya-ku, Tokyo 156-8506, Japan; ^3^Tsumura Research Laboratories, Tsumura & Co., 3586 Yoshiwara, Amimachi, Inashiki, Ibaraki 300-1192, Japan; ^4^Department of Anatomy, School of Medicine, Keio University, 35 Shinanomachi, Shinjuku-ku, Tokyo 160-8582, Japan

## Abstract

The aging-induced decrease in axonal myelination/remyelination is due to impaired recruitment and differentiation of oligodendrocyte progenitor cells (OPCs). Our previous studies have shown that a monoclonal antibody to DEAD (Asp-Glu-Ala-Asp) box polypeptide 54 (Ddx54), a member of the DEAD box family of RNA helicases, (1) specifically labels oligodendrocyte lineages, (2) binds to mRNA and protein isoforms of myelin basic proteins (MBP), and (3) regulates migration of OPCs from ventricular zone to corpus callosum in mice. It has also been demonstrated that specific loss of a 21.5 kDa MBP isoform (MBP21.5) reflects demyelination status, and oral administration of an extract of Chinpi, citrus unshiu peel, reversed the aging-induced demyelination. Here, we report that Chinpi treatment induced a specific increase in the MBP21.5, led to the reappearance of Ddx54-expressing cells in ventricular-subventricular zone and corpus callosum of aged mice, and promoted remyelination. Treatment of* in vitro* OPC cultures with Chinpi constituents, hesperidin plus narirutin, led to an increase in 5-bromo-2′-deoxyuridine incorporation in Ddx54-expressing OPCs, but not in NG2- or Olig2-expressing cell populations. The present study suggests that Ddx54 plays crucial role in remyelination. Furthermore, Chinpi and Chinpi-containing herbal medicines may be a therapeutic option for the aging-induced demyelination diseases.

## 1. Introduction

Myelin is the coiled cell membrane that insulates the axons of nerve fibers. In the central nervous system (CNS), myelin is synthesized by oligodendrocytes, and in rodents, the majority is produced during the first six postnatal weeks after proliferation of oligodendrocyte progenitor cells (OPCs). OPCs persist in the brain parenchyma of the adult mouse CNS, comprising approximately ~5% of all neural cells [1]. Regeneration of the myelin sheath (remyelination) occurs as a spontaneous response to neuronal demyelination. Parenchymal OPCs continue to divide and differentiate into myelinating oligodendrocytes throughout life [2], playing a crucial role in the repair process [3]. Neural stem cells (NSCs) within the ventricular-subventricular zone [4–7] also maintain their ability to generate oligodendrocytes and promote remyelination [8, 9]. However, similar to other body repair processes, remyelination becomes less efficient with age [10–12].

There is a general consensus that CNS remyelination involves the recruitment and differentiation of OPCs, which facilitates the development of new oligodendrocytes [6, 13, 14], rather than to new processes formation by previously myelinating oligodendrocytes [3, 15]. In response to demyelination, OPCs are recruited to the lesion site, followed by proliferation, migration, and rapid differentiation in the demyelinated area. A key factor that causes insufficient remyelination, in various demyelinating diseases as well as aging-induced demyelination, is deficient OPC proliferation [10, 16]. Several growth factors affect the* in vitro* proliferation, migration, and differentiation of OPCs [17], including platelet derived growth factor *α* and fibroblast growth factor-2, both of which promote the proliferation and motility of adult OPC [18, 19]. However, the molecules and mechanisms that drive OPCs recruitment after demyelination remain largely unclarified.

In a previous study, we reported that a monoclonal antibody 4F2 recognizes Ddx54, a member of the DEAD-box protein family that specifically stains oligodendrocyte lineages at the early embryonic stage through to adulthood. Ddx54 is expressed in neural tube cells at the earliest stage of oligodendrocyte lineage development (e.g., E 9.5 in rats), before they migrate away from the subventricular zone, suggesting that Ddx54 may be implicated [20]. Accordingly, a pilot study demonstrated that adenoviral vector-mediated knockdown of Ddx54 expression inhibited translocation of OPCs from the subventricular zone to the emerging white matter in mice [21]. Interestingly, Ddx54 protein interacted with both the mRNA and protein forms of myelin basic protein (MBP) [20, 21]. A 21.5-kDa isoform of phosphorylated MBP, which was closely related to myelination status, was identified in lipid-rafts of myelin, while the absence of this isoform corresponded to cuprizone- and aging-induced demyelination [22, 23] as well as Ddx54 knockdown [21].

In another project investigating the potential efficacy of herbal medicines for demyelinating diseases, Chinpi, which is derived from the peel of citrus fruit unshiu [24], and Chinpi-containing Kampo (traditional Japanese) medicine [22] inhibited both cuprizone- and aging-induced demyelination via an FcR*γ*-Fyn signaling cascade.

In this study, the* in vivo* effect of Chinpi on demyelination and Ddx54-expressing oligodendrocyte linages was investigated in the ventricular-subventricular zone of the lateral ventricles and the corpus callosum of aged mice and the* in vitro* effects were examined using OPC cultures.

## 2. Materials and Methods

### 2.1. Mice

Twenty-eight-month-old C57BL/6 mice were supplied by the department of Animal science, Tokyo Metropolitan Institute of Gerontology, and acclimated for one week in the animal experimental research laboratory and then randomly divided into two groups of five mice each. Mice were placed individually in plastic cages and subjected to a 12 hr light/12 hr dark cycle at 24 ± 1°C temperature and 55 ± 5% relative humidity. Mice had access to food and water* ad libitum*. Chinpi was administered by dissolving the extract in drinking water at 0.5% weight/volume [22, 24]. The mean amount of Chinpi consumed via drinking water was 4.1 mL ± 0.2 mL/day/mouse. The mice received Chinpi for two months, from 28 to 30 months of age. All animal experiments were performed with the approval of the guidelines of the Institutional Animal Care and Use Committee of Keio University School of Medicine (approval number 08071).

### 2.2. Drugs and Reagents

Dried extracts made from dried peels, the citrus fruit unshiu, were prepared by Tsumura & Co. (Tokyo Japan). In brief, approximately 20 g of the dried peel was immersed in 200 mL of distilled water and heated to boiling point. After 1 h at 100°C, followed by cooling to room temperature, the extracts were filtered and lyophilized to obtain the dried extract. The extracts dried extracts were subsequently examined for contents of flavonoids and used for* in vitro* and* in vivo* studies. Three-dimensional profiles of the chemical compounds detected within Chinpi extract were reported in a previous paper [24]. Hesperidin, narirutin (two major components of Chinpi extract), and other reagents were purchased from Wako Pure Chemical Industry, Ltd. (Osaka, Japan) unless otherwise stated.

### 2.3. Immunohistochemistry

All mice were deeply anesthetized with ether and perfused with 4% paraformaldehyde and stored in a fixative solution containing 0.2% glutaraldehyde in 0.2 M phosphate buffer (pH 7.4). The cerebral hemispheres were separated and serial 10 *μ*m coronal sections were cut on a microtome. Immunohistochemistry was performed as previously described [25, 26]. Free-floating sections were initially rinsed in 20 *μ*M phosphate-buffered saline (PBS) and incubated in a mixture of 3% hydrogen peroxide and 0.1% TritonX-100 for 15 min at room temperature. After rinsing in 20 mM PBS, the sections were incubated overnight at 4°C with the primary antibody (4F2 or anti-MBP, see below) which was diluted with 20 mM PBS-containing 0.5% skimmed milk. After rinsing in 20 mM PBS for 15 min, sections were incubated with biotinylated secondary antibody (1 : 100, Vector Laboratories) for 30 min at 37°C. The sections were rinsed with 20 mM PBS for 15 min and subsequently incubated in avidin-biotin peroxidase complex (Vectastain ABC kit, Vector Laboratories) for 30 min at 37°C. After rinsing in 20 mM PBS, immunoreactions were visualized in a solution containing 0.01% diaminobenzidine tetrahydrochloride and 0.01% hydrogen peroxide in 50 mM Tris buffer (pH 7.4) at 37°C for 5 to 10 min. The nuclei were counterstained with hematoxylin according to the manufacturer's instructions. Sections were mounted on MAS-coated glass slides (Matsunami Glass, Osaka, Japan), air-dried on a hot plate at 40°C, and coverslipped with Entellan New (Merck, Darmstadt, Germany) after dehydration with ethanol and xylene. The primary antibodies, anti-Ddx54 mouse monoclonal antibody 4F2 [21] (1 : 200) and anti-MBP rabbit polyclonal antibody [27], (1 : 500) were prepared in our laboratory.

### 2.4. Toluidine Blue Staining

Mice were perfused with 4% paraformaldehyde and the brains were fixed overnight in an acid-alcohol solution (95% ethanol/5% acetic acid, v/v). The brain tissues were embedded in paraffin and sliced into 10 *μ*m thick sections. The sections were then mounted and stained for myelin using Luxol® Fast Blue (LFB: Acros Organics, Fair Lawn, NJ, USA) solution (0.1% LFB in 95% ethanol containing 0.05% acetic acid) and stained overnight at 60°C. Toluidine blue staining was performed according to the manufacturer's protocol (Wako Pure Chemical Industry).

### 2.5. Electron Microscopy

Each cerebrum was fixed with 2.5% glutaraldehyde and then postfixed with 1% OsO_4_. After dehydration in ethanol, the specimens were embedded in Quetol 812 (Nisshin EN, Tokyo, Japan) and ultrathin sections stained with 2% uranylacetate and lead solution were observed using a JOEL 100C electron microscope (JOEL, Tokyo, Japan), as described elsewhere. For the *G*-ratio measurement (the ratio of the axon diameter to the diameter of the axon plus the surrounding myelin), at least three mice per group were used. Between 8 and 10 microphotographs at a high magnification (×5000 or ×10000) of the coronal sections of the corpus callosum at the midline were taken for each mouse, and the *G*-ratios of at least 90 axons were measured. Axons with aberrant morphology, including myelin reduplication, abnormal splitting of the myelin sheath, vacuolization of the myelin lamellae, and myelin balloon formation, were excluded because these types of aberrant myelin morphology make it difficult to accurately evaluate the degree of myelination. For example, the extraordinarily large myelin sheath of axons with an abnormal morphology arising from the repetition of unaccomplished remyelination should not be estimated in the same manner as the myelin sheaths of normal axons. An additional quantitative study was undertaken to assess the number of myelinated fibers per 400 *μ*m^2^ using three mice per group.

### 2.6. Preparation of Purified OPCs

The method of Seiwa et al. was used for mouse OPC culture, as described [28]. After washing with Dulbecco's modified Eagle's medium (DMEM, Invitrogen Carlsbad, CA), cerebral hemispheres from E 17 mice were enzymatically dissociated in a mixture of 0.3% dispase II and 0.05% DNase (Roche Molecular Biochemicals, Mannheim, Germany) in DMEM. The dissociated cells were sieved through a 70 *μ*m pore nylon mesh (number 2350, Becton Dickson, Franklin, NJ) and then seeded on poly-*L*-lysine (PLL, ICN) coated 10 cm diameter culture dishes (Greiner Bio One, Tokyo, Japan) at a density of 1.3 × 10^7^ cells/dish in DMEM containing 10% FBS. After 5 days of culture, the cells were passaged with 0.2% trypsin in PBS and centrifuged for 10 min at 1,000 rpm at 4°C in a 15 mL tube. The cells were resuspended in a serum-free medium (“BS” medium) consisting of DMEM supplemented with glucose (5.6 mg/mL), kanamycin (60 mg/mL), insulin (5 *μ*g/mL), transferrin (0.5 *μ*g/mL), BSA (100 *μ*g/mL), progesterone (0.06 ng/mL), putrescine (16 *μ*g/mL), sodium selenite (40 ng/mL), and thyroxine (40 ng/mL) and incubated for 2 hr at 37°C in a CO_2_ incubator at a density of 2.5 × 10^6^ cells/mL. These procedures are essential to eliminate neurons, microglia, and astrocytes. The cells were then thoroughly resuspended in serum-free medium and cultured at a density of 2 × 10^6^/mL cells on a noncoated, 10 cm dish for 2 days. Immunocytochemistry demonstrated that more than 95% of the cultured cells were routinely determined to be positive for both OPC markers, Olig2 (1 : 200, goat polyclonal; R&D Systems, Minneapolis, MN) and NG2 (1 : 200; rabbit polyclonal, a gift from Dr. William Stallcup, Bumham Institute for Medical Research, La Jolla, CA) by immunocytochemistry.

### 2.7. 5-Bromo-2′-deoxyuridine (BrdU) Incorporation

The cells were pulsed with 20 *μ*M BrdU (Sigma Aldrich) for 48 hr, fixed with 70% ethanol for 30 min at 22°C, rinsed with 0.05% BSA in PBS, and then treated with 2 N HCl for 10 min at room temperature to denature the DNA in the preparation. The sample was then treated with 0.1 M sodium tetraborate (pH 8.5) to neutralize HCl. Cells were incubated with a mouse IgG antibody to BrdU (1 : 300; Dako, Glostrup, Denmark) for 2 h and incubated with the FITC-conjugated goat anti-mouse IgG secondary antibody (1 : 300) for 90 min at room temperature. Cell cycle analysis was performed using Cycletest Plus DNA reagent kit (Becton Dickson) according to the supplier's protocol.

### 2.8. Western Blot Analysis

Western blot analysis was performed as described previously [28]. Samples (brain homogenate, cell lysate) were separated using SDS-PAGE (4–20% gel gradient) for 2 h and then prepared for immunoblotting by semi-dry transfer onto an Immobilon PVDF membrane (Millipore, Bedford, MA, USA). The membranes were blocked with 5% skimmed milk (Difco Laboratories, Detroit, MA, USA) in Tris-buffered saline (pH 7.6) for 1 hr, and the blots were incubated with 4F2, anti-MBP or anti-*β*-actin (Sigma Aldrich, St. Louis, MO) antibodies for 2 hr. Next the membranes were washed three times for 10 min each using 1% skimmed milk in Tris-buffered saline, incubated with alkaline phosphatase-conjugated goat anti-mouse or anti-rabbit IgG (diluted 1 : 800) for 2 h, and developed using nitro blue tetrazolium and 15-bromo-4-chloro-3-indolyl-phosphate solution (both from Sigma, St Louis, MO, USA).

### 2.9. Statistics

Morphological data are represented as mean ± SEM. Differences among groups were analyzed using Student's *t*-test. *P* < 0.05 is considered statistically significant.

## 3. Results

### 3.1. Effect of Chinpi on Aging-Induced Demyelination

**Figure 1:**
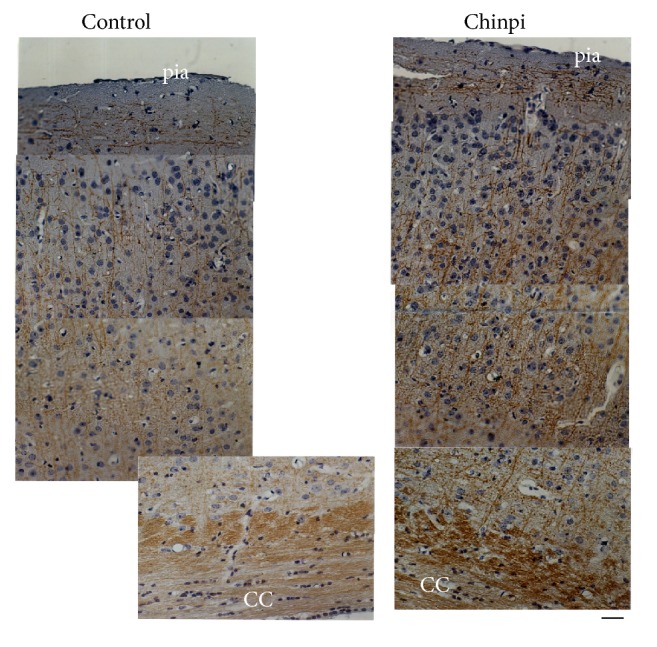
Immunohistochemistry of coronal section of the cerebrum using anti-MBP antibody. Twenty-eight-month-old mice were treated with or without Chinpi in drinking water for 2 months. An overview of the gray and white matter at 30 months old is shown. Myelin stained with anti-MBP antibody (brown signals) reveals the vertical, myelinated nerve fibers running from the corpus callosum (CC) to the pia matter. The number of myelinated nerve fibers was increased after two months of Chinpi, and age-induced demyelination was reduced compared with controls. Scale bar = 28 *μ*m.

**Figure 2:**
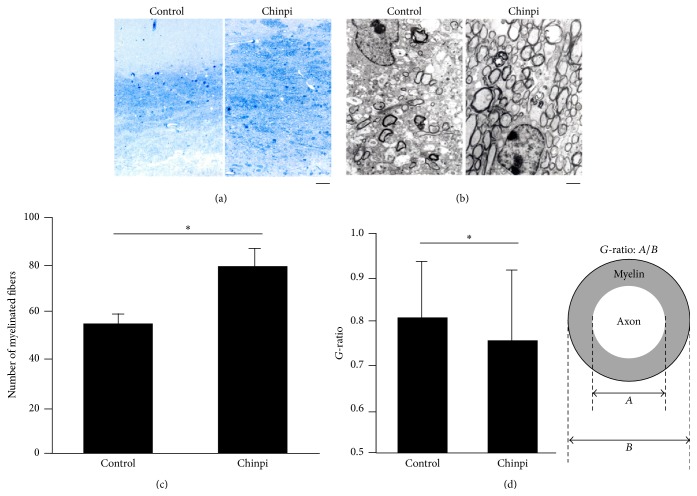
(a) Toluidine blue staining of cross sections of the corpus callosum. Myelin staining was increased in Chinpi-treated mice. Scale bar = 85 *μ*m. (b) Electron micrographs of cross section of the corpus callosum show aging-induced demyelination in control mice. The myelination status was improved after two months of Chinpi treatment. Scale bar = 2 *μ*m. (c) The density of myelinated fibers per 300 *μ*m^2^. Treatment with Chinpi led to a significant increase in the myelinated nerve fiber density. The number of myelinated fibers was counted using five electron micrograph images from each mouse. Data represent mean ± SEM (*n* = 3): ^*∗*^ *P* < 0.05. (d) The *G*-ratio, an indicator of demyelination, which is defined as the ratio of the axon diameter to that of the axon diameter plus the surrounding myelin sheathe (see right panel for formula) was calculated. The increased *G*-ratio in the elderly mice was improved by Chinpi treatment. The number of measured axons was 90, using eight electron micrographs from three mice. Data represent mean ± SEM. ^*∗*^ *P* < 0.05 versus control. The analyses were performed using 30-month-old mice.

After the two-month administration period, the gray and white matter of the aged 30-month-old mice in the control and Chinpi groups were examined. Immunohistochemistry with the anti-MBP antibody stained the corpus callosum to the pia mater, but immunoreactivity was more intense in the Chinpi-treated mice than the age-matched control mice (Figure 1). Observation of myelinated axons by toluidine blue staining (Figure 2(a)) and electron microscopy (Figure 2(b)) revealed the presence of aging-induced demyelination in the control mice, while remyelination was stimulated in Chinpi-treated mice. Quantitative analyses of the numbers of myelinated fibers (Figure 2(c)) and *G*-ratio (Figure 2(d)) demonstrated that both the number of myelinated axons and the thickness of the myelin sheath were increased in Chinpi-treated mice, presumably because of enhanced remyelination. Similar to previous reports using extremely old rodents (31 months), the brains of untreated aged mice showed many abnormalities in the myelin sheath, including myelin balloon formation, splitting of the myelin sheath, and vacuolization of myelin lamellae [29]. In contrast, a marked reduction in abnormal histological findings was observed in the brain of Chinpi-treated mice. These results are consistent with our previous research using mice fed on a diet containing 1% Chinpi for two months [24].

### 3.2. Effect of Chinpi on Ddx54-Expressing OPCs

**Figure 3:**
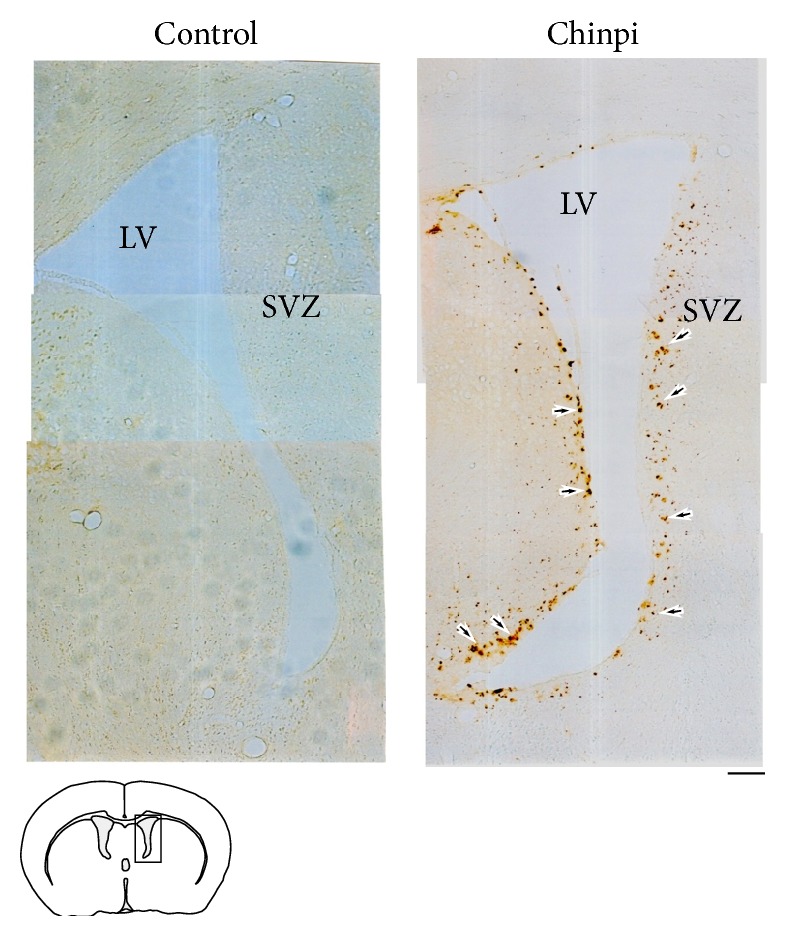
Immunohistochemistry of sections of the ventricular (VZ) and subventricular zone (SVZ) of the lateral ventricles (LV) with anti-Ddx54 antibody. Ddx54 immunoreactivity was negligible in the control specimens. Strong Ddx54 immunoreactivity but with an uneven distribution was observed in Chinpi-treated brains. Scale bar = 28 *μ*m. The analyses were performed using 30-month-old mice.

**Figure 4:**
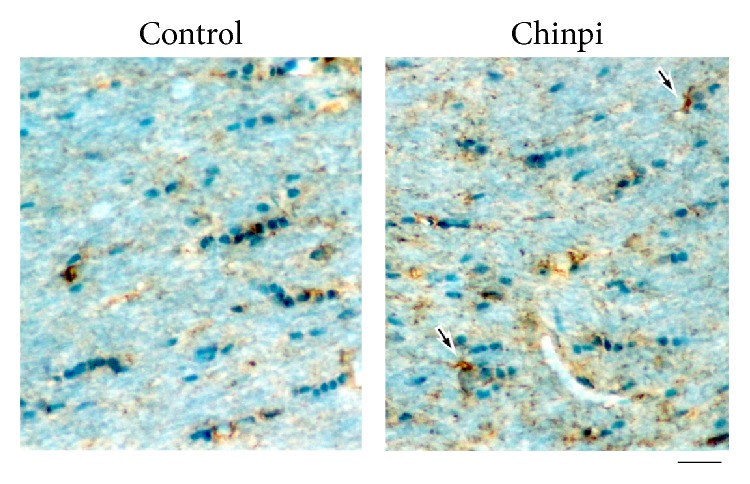
Immunohistochemistry of corpus callosum sections stained with anti-Ddx54 antibody. In Chinpi-treated mice, Ddx54-expressing cells with the morphology of immature/mature oligodendrocytes (arrows) were observed, while in the control mice such cells were scarcely detected. Scale bar = 112 *μ*m. This experiment was performed using 30-month-old mice.

**Figure 5:**
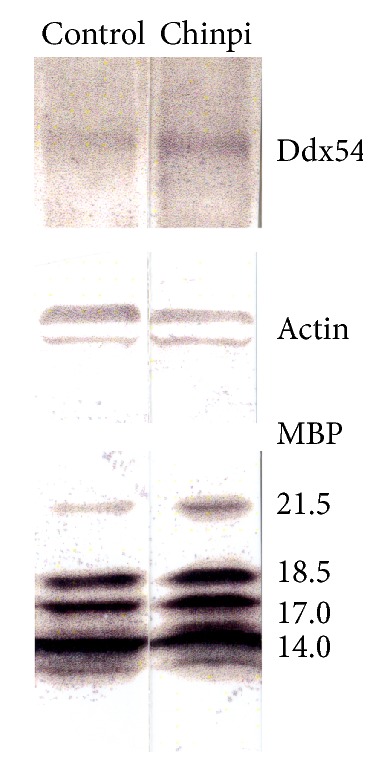
Western blotting of brain homogenates from control and Chinpi-treated aged mice (at 30 months old). The signal intensity of both Ddx54 protein and 21.5-kDa isoform of MBP was higher in Chinpi-treated mice compared with control mice.

The effect of Chinpi on Ddx54-expressing cells in the area of ventricular zone region facing the side of the lateral ventricles was investigated. Ddx54 immunoreactivity was scarcely detected in the control elderly (both 28- and 30-month old) mice as shown in Supplementary Fig. S1 in Supplementary Material available online at http://dx.doi.org/10.1155/2016/8692698 and Figure 3, respectively. Surprisingly, in Chinpi-treated mice, clear Ddx54 immunoreactivity was detected in the medial and lateral wall of the lateral ventricles at the most anterior and posterior regions (Figure 3). This pattern of Ddx54 labeling coincided with that of A2B5-positive cells in the same region of young to middle-aged mice [30, 31] as well as that of Ddx54-positive cells in younger mice [20], which represent OPC in this region. The appearance of Ddx54-expressing cells with the morphology of immature/mature oligodendrocytes was also observed in the corpus callosum (Figure 4). Accordingly, immunoblot analysis also indicated that the level of Ddx54 protein was higher in the brain homogenate of Chinpi-treated mice (Figure 5). The specific decrease in 21.5 kDa isoform of MBP (MBP21.5) in the aged mice, which is known to be closely related to the demyelination status [22, 32], was also improved in the Chinpi-treated mice (Figure 5).

### 3.3. Effect of Chinpi Compounds on OPC Proliferation* In Vitro*

**Figure 6:**
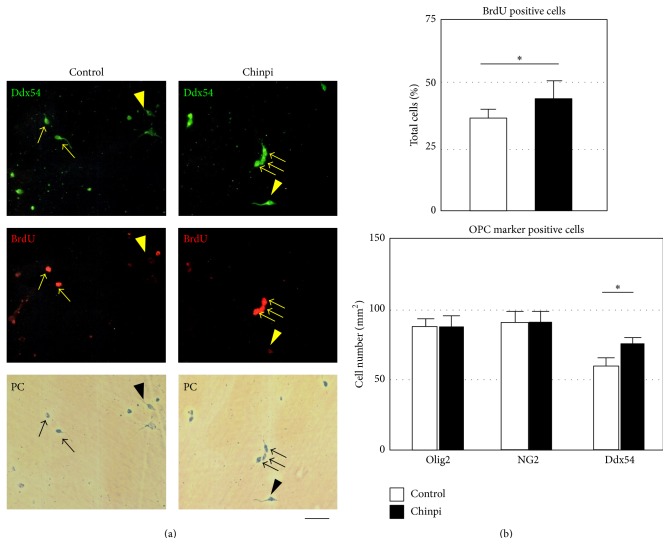
(a) Immunofluorescence staining of purified OPC cultures with antibodies against Ddx54 and BrdU. At 48 hr after addition of 20 *μ*M BrdU, cells cultured with or without hesperidin plus narirutin (10 *μ*g/mL each) were stained with anti-Ddx54 (top row, green fluorescence) and anti-BrdU antibodies (middle row, red fluorescence). Ddx54-positive cells (arrows in top row), BrdU-positive cells (arrows in middle row), and phase contrast images (bottom row) were shown. Arrowhead indicates BrdU-negative cells. Scale bar = 20 *μ*m. (b) Quantitation of proliferating OPCs by double immunofluorescence staining of purified OPC cultures with antibodies for OPC markers and anti-Ddx54 antibody. Top row: percentage of BrdU^+^ cells of total cells. Bottom row: the number of the cells coexpressing BrdU and various OPC markers. Selective proliferation of Ddx54^+^ cells, but not in Olig2^+^ or NG2^+^ cells, was induced by Chinpi ingredients resulting in an increase in the total number of cultured cells. Data are represented as mean ± SEM. ^*∗*^ *P* < 0.05 versus control.

The effect of two specific compounds of Chinpi (hesperidin plus narirutin) was investigated at the cellular levels using* in vitro* OPC cultures (Figure 6(a)). Purified OPCs were incubated with Chinpi components (10 *μ*g/mL hesperidin plus 10 *μ*g/mL narirutin) and cell proliferation was assessed after 48 h by double immunostaining for BrdU incorporation (a proliferation marker) and Ddx54 antibody. As shown in Figure 6(b), the addition of Chinpi ingredients increased the ratio of BrdU-positive cells, and the increase was due to the proliferation of Ddx54^+^ OPCs, not Olig2^+^ OPCs or NG2^+^ OPCs.

## 4. Discussion

Aging is a primary factor that impairs remyelination, as observed in neurodegenerative diseases such as Alzheimer's disease in which patients develop progressive cognitive decline. Cells of the oligodendrocyte lineage, specifically OPCs, may be targeted as source of remyelination because they are present in the adult brain parenchyma of not only mice but also humans [22, 32]. Neural stem cells in the adult subventricular zone of the lateral ventricles may also represent an alternative source of OPCs for remyelination [33]. Therefore it is possible that the promotion of proliferation, recruitment, and differentiation of OPCs in the lesion site is a promising therapeutic strategy to repair myelin deficits. Only a few factors are known to induce proliferation, differentiation, and motility in OPCs [34, 35].

**Figure 7:**
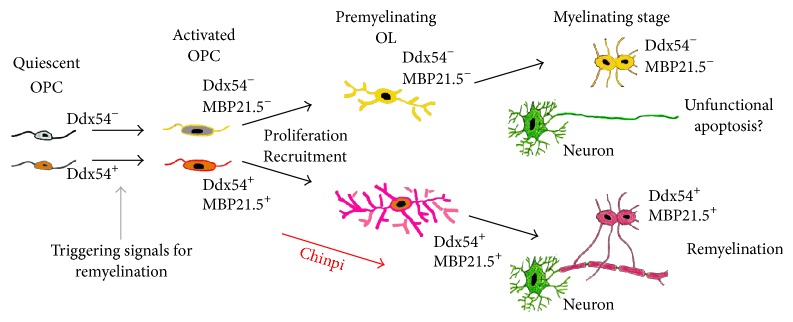
A hypothesis of the fate of oligodendrocyte linages in remyelination. Demyelination triggers the proliferation and recruitment of OPC. Ddx54^+^ OPC differentiate into MBP21.5-expressing oligodendrocytes (OL) that obtain myelinating capacity, while Ddx54^−^ OPC differentiate into MBP21.5^−^ OL that cannot myelinate axons.

The present study demonstrated that two months of treatment with a water-soluble extract of Chinpi, administered via the drinking water, reversed aging-induced demyelination in mice (Figure 1). Furthermore, Chinpi was associated with an increase in Ddx54-expressing cells in the ventricular facing side of the lateral ventricle and corpus callosum (Figures 3 and 4). Ddx54 is considered to be a specific marker of the oligodendrocyte linages from the embryonic stage to adulthood [20], and it is indispensable for both the OPC maturation (i.e., production of MBP21.5) and translocation from the ventricular zone to the corpus callosum [21]. Furthermore, close relationships between expression levels of Ddx54 and MBP21.5 and myelination status have been demonstrated in different demyelination models (cuprizone, aging and FcR*γ*/Fyn double knockout) [21, 23, 36]. This suggests that the Ddx54-expressing cells observed in the present study may represent newly generated OPCs, which are destined to be myelinating oligodendrocytes for efficient remyelination (Figure 7).

Of note, Chinpi did not attenuate the loss of Ddx54-expressing OPCs during the two-month treatment period until the mice were 30 months old but actively increased the number of Ddx54-expressing cells. This was demonstrated by the observation that Ddx54 immunoreactivity was negligible in both the 28-month-old control mice and the 30-month-old control mice (Supplementary Fig. S1). Although NSCs are found in the subventricular zone of the aged brain, their proliferative and neurogenic capacity are diminished with age [37]. However, the reappearance of Ddx54-expressing cells in the subventricular zone following Chinpi treatment suggests that a proportion of NSCs may be capable of reactivation to produce new oligodendrocyte lineages, presumably leading to remyelination. In accordance,* in vitro* experiments with hesperidin and narirutin (two of the active constituents of Chinpi) revealed a significant increase in the ratio of BrdU incorporation, indicative of cell proliferation into Ddx54^+^ cells (Figure 4). The present findings strongly suggest that reactivation of “resting” NSC in the aged brain, as exemplified by Chinpi, may be a promising strategic option for the treatment of aging-induced demyelination.

Ddx54 is a specific marker for oligodendrocytes lineages, including OPCs; however, Ddx54 expression is not expressed in all Olig2^+^ and/or NG2^+^ OPCs (Supplementary Fig. S2). Similarly, only a subset of O1^+^ immature, O4^+^ immature, and/or MBP^+^ mature oligodendrocytes express Ddx54 protein [20]. Many OPCs are generated in the brain; however, only a small proportion of these differentiate and attain the ability myelinate axons. The vital molecules necessary for maturation of OPCs into myelinating oligodendrocytes are largely unknown, but Ddx54 may be a candidate molecule (Figure 7). Ddx54 binds to both the mRNA and protein of MBP isoforms: binding to mouse MBP mRNA was previously demonstrated [20] and binding to mouse and rat MBP proteins is shown in supplementary Fig. S3 and the previous report [20], respectively. Ddx54 is a DEAD box RNA-helicase that is implicated in the key steps of RNA-related processes, including transcription, pre-mRNA processing, ribosome biogenesis, RNA export, translational initiation, and RNA degradation [16, 38]. Our previous studies demonstrated that Ddx54 knockdown inhibits the intrusion of OPCs from the ventricular zone to the corpus callosum, resulting in the failure of axonal myelination. We have also reported that oligodendrocytes cultured under oxygen-glucose deprivation lose the ability to interact with neuron and also display reduced expression of Ddx54 and MBP21.5 [36]. These findings address the possibility that Ddx54 may play a critical role in MBP mRNA transport, “on-site” synthesis of MBP protein in the myelin sheath, and therefore axonal contact. To fully elucidate the biological roles in Ddx54, extensive analyses using transgenic and/or knockout Ddx54 models are necessary. Such studies are currently underway.

## 5. Conclusion

The present findings suggest a novel therapeutic strategy for the aging-induced decline in the efficiency of remyelination. Chinpi and Chinpi-containing Japanese traditional (Kampo) medicines are widely used in clinics in Japan. In particular, Kampo medicines of pharmaceutical grade are manufactured under strict scientific control of active ingredients and impurities and integrated in national insurance systems resulting in the widespread use of Kampo medicines in almost all national, public, and university hospitals as well as private clinics. These medicines may provide a readily available means for the treatment of various demyelinating diseases. Furthermore, better elucidation of the mechanisms by which Chinpi promotes remyelination will greatly contribute to the scientific understanding of the biological processes underlying myelination and remyelination.

## Supplementary Material

Supplementary Figure S1: Immunohistorchemistry of the sections of ventricular and subventricular zone (V-SVZ) with anti-Ddx54 antibody.
Supplementary Figure S2: Double immunofluorescence staining of purified OPC cultures with antibodies against Olig2 or NG2 and Ddx54.
Supplementary Figure S3: Coimmunoprecipitation of the Ddx54 antigen with isoforms of MBP in thirty-month-old mouse brain homogenates.

## Abbreviations

BrdU: 5-Bromo-2′-deoxyuridine
CNS: Central nervous system
LV: Lateral ventricles
MBPs: Myelin basic proteins
NSCs: Neural stem cells
OPCs: Oligodendrocyte progenitor cells
SVZ: Subventricular zone
V-SVZ: Ventricular-subventricular zone
VZ: Ventricular zone.

## Highlights

The highlights are as follows:

- Chinpi (citrus peel) reverses aging-induced demyelination.
- Chinpi increased Ddx54-expressing OPCs* in vivo* and* in vitro*.
- Ddx54^+^ 21.5 kDa MBP^+^ OPC may be critical for myelination/remyelination.
