# Supplementary material for: An Extract of Chinpi, the Dried Peel of the Citrus Fruit Unshiu, Enhances Axonal Remyelination via Promoting the Proliferation of Oligodendrocyte Progenitor Cells
Source: Evid Based Complement Alternat Med. 2016 Feb 28;2016:8692698. doi: 10.1155/2016/8692698 (PMC4789069; doi:10.1155/2016/8692698)

Supplementary Fig. S1

Immunohistorchemistry of the sections of ventricular and subventricular zone (V-SVZ) sections of the lateral ventricles (LV) of twenty-eight-month old mouse with anti-Ddx54 antibody. V-SVZ sections from the LV of elderly mice were stained with 4F2 antibody but no signal was detected.


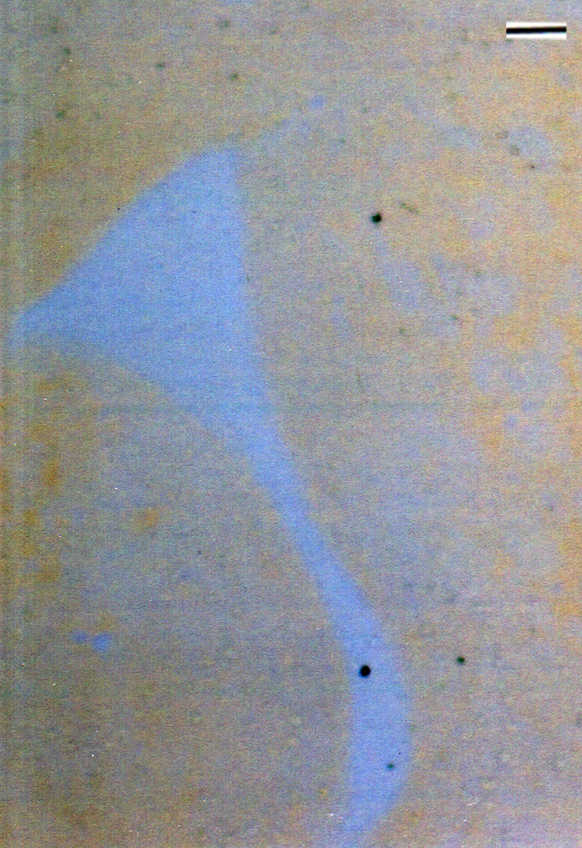


Supplementary Fig. S2

Double immunofluorescence staining of purified OPC cultures with antibodies against Olig2 or NG2 and Ddx54. The cultured OPCs were stained with anti-Ddx54 (top row, green fluorescence) and anti-Olig2 or anti0NG2 antibodies (middle row, red fluorescence). Ddx54-positive cells (arrows in top row), BrdU-positive cells (arrows in middle row), phase-contrast images (bottom row) are shown. Arrowhead indicates Ddx54-negative, Olig2 or NG2-positive cells. Scale bar = 20µm.


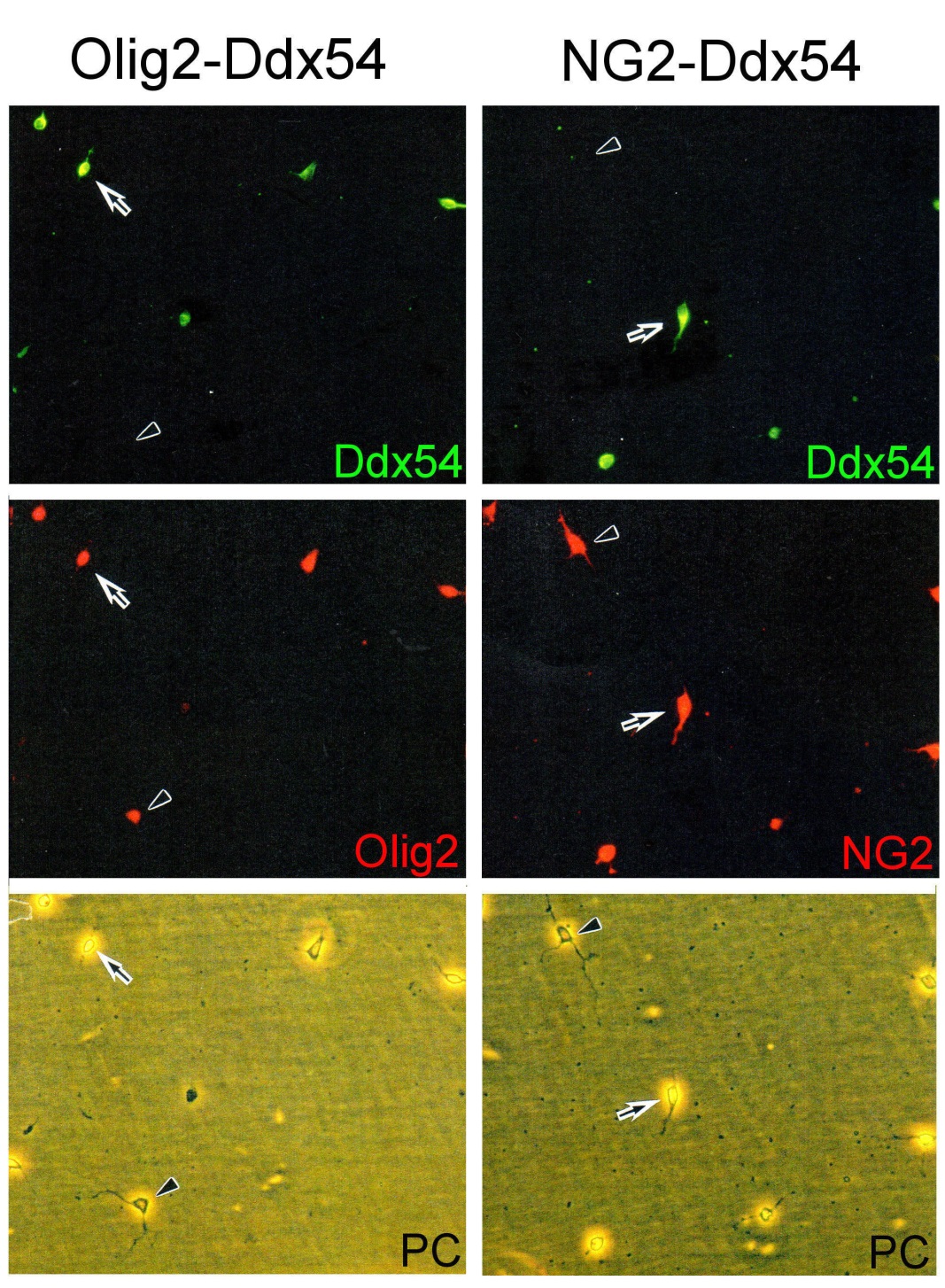


Supplementary Fig. S3

Coimmunoprecipitation of the 4F2 antigen with isoforms of MBP in thirty-month-old mouse brain homogenates. Mouse brain was lysed and centrifuged, and the supernatant was immunoprecipitated with the 4F2 antibody. The standard purified mouse MBP (A), the supernatant before immunoprecipitation (B) and the immunoprecipitate pellet redissolved in lysis buffer (C) were electrophoresed, blotted, and detected by anti-MBP antibody. The isoforms of MBP were coimmunoprecipitated with 4F2 antibody, suggesting an association between the 4F2 antigen and isoforms of MBP in mouse brain. Although all four MBP isoforms precipitated, the 17.0- and 21.5-kDa isoforms seemed to be recovered more efficiently than the 14.0- and 18.5-kDa isoforms.


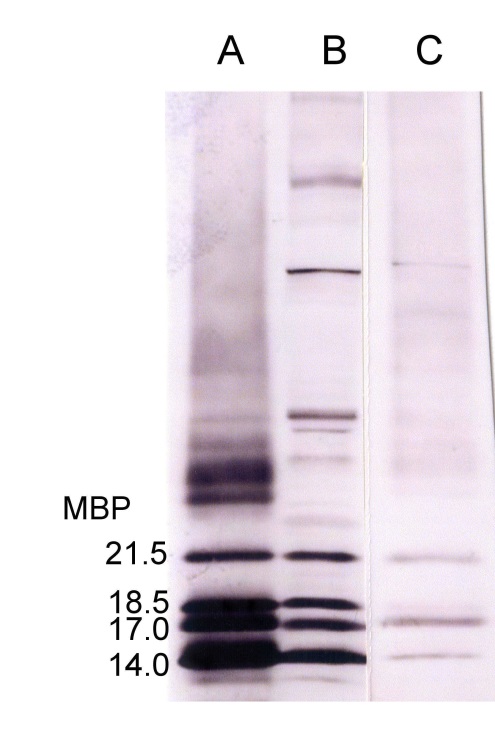

Supplement: Supplementary file 1 — Supplementary Figure S1: Immunohistorchemistry of the sections of ventricular and subventricular zone (V-SVZ) with anti-Ddx54 antibody. Supplementary Figure S2: Double immunofluorescence staining of purified OPC cultures with antibodies against Olig2 or NG2 and Ddx54. Supplementary Figure S3: Coimmunoprecipitation of the Ddx54 antigen with isoforms of MBP in thirty-month-old mouse brain homogenates. [file 8692698.f1.docx]
